# Interventional MRI-Guided Ablation of Left Ventricular Tachycardia

**DOI:** 10.1161/CIRCULATIONAHA.126.080380

**Published:** 2026-04-14

**Authors:** Luuk H.G.A. Hopman, Marco J.W. Götte, Jules L. Nelissen, Raschel D. van Luijk, Marjolein F. Koster, Mika J. Sybrandi, Katherine Lindborg, Pieter G. Postema, Steven A.J. Chamuleau, Cornelis P. Allaart, Michiel J.B. Kemme

**Affiliations:** 1 Department of Cardiology, Amsterdam UMC, Amsterdam, The Netherlands (L.H.G.A.H., M.J.W.G., M.F.K., M.J.S., P.G.P., S.A.J.C., C.P.A., M.J.B.K.).; 2 Division of Cardiology, Department of Cardiac Sciences, Cumming School of Medicine, Libin Cardiovascular Institute, University of Calgary, Calgary, Canada (M.J.W.G.).; 3 Department of Radiology and Nuclear Medicine, Amsterdam UMC, Amsterdam, The Netherlands (J.L.N., R.D.V.).; 4 Imricor Medical Systems, Burnsville, MN (K.L.).; 5 Department of Cardiology, Radboud UMC, Nijmegen, The Netherlands (C.P.A.).

Catheter ablation is a well-established therapy for drug-refractory ventricular tachycardia (VT) in ischemic cardiomyopathy.^1^ Conventional workflows rely on fluoroscopy and electroanatomical mapping, which provide limited anatomical detail and identify arrhythmogenic substrate indirectly through voltage-based surrogates rather than direct tissue characterization.

Cardiovascular magnetic resonance imaging (MRI) enables high-resolution assessment of myocardial structure, including 3-dimensional (3D) late gadolinium enhancement (LGE) imaging for substrate definition. Interventional cardiovascular MRI allows real-time catheter navigation without ionizing radiation and permits integration of anatomical and tissue information during the procedure. While real-time magnetic resonance–(MR) guided ablation has been reported for atrial arrhythmias and premature ventricular complexes, fully MR-guided left-sided VT ablation has not been described.^2,3^ We report the workflow and short-term outcome of a first-in-human interventional cardiovascular MRI VT ablation.

## PATIENT HISTORY

A 79-year-old man with ischemic cardiomyopathy, an MR-compatible dual-chamber implantable cardioverter defibrillator (ICD), and recurrent monomorphic VT despite 2 previous fluoroscopy-guided endocardial ablations and amiodarone was enrolled as a roll-in case in the prospective VISABL-VT trial (Vision-MR Ablation Catheter 2.0 for Treatment of Ventricular Tachycardia; NCT05543798). Patient selection was guided by study predefined inclusion and exclusion criteria. The study was approved by the institutional review board, and the patient provided written informed consent for participation and publication. The data underlying this article are not available for sharing because of the ongoing trial.

## PREPROCEDURAL MRI

Preprocedural MRI (1.5-T) demonstrated a left ventricular ejection fraction of 32% and inferolateral wall thinning. A contrast-enhanced high-resolution 3D MR-angiogram was acquired for whole-heart segmentation and visualization of ICD leads, allowing planning of transseptal sheath advancement along the septal aspect of the leads (Figure [A]).^4^ Two-dimensional wideband LGE imaging demonstrated inferoposterolateral infarction without device-related artifact from the left pectoral ICD affecting image interpretation. A high-resolution 3D LGE data set was subsequently acquired and segmented for substrate characterization (Figure [B]).

**Figure.**
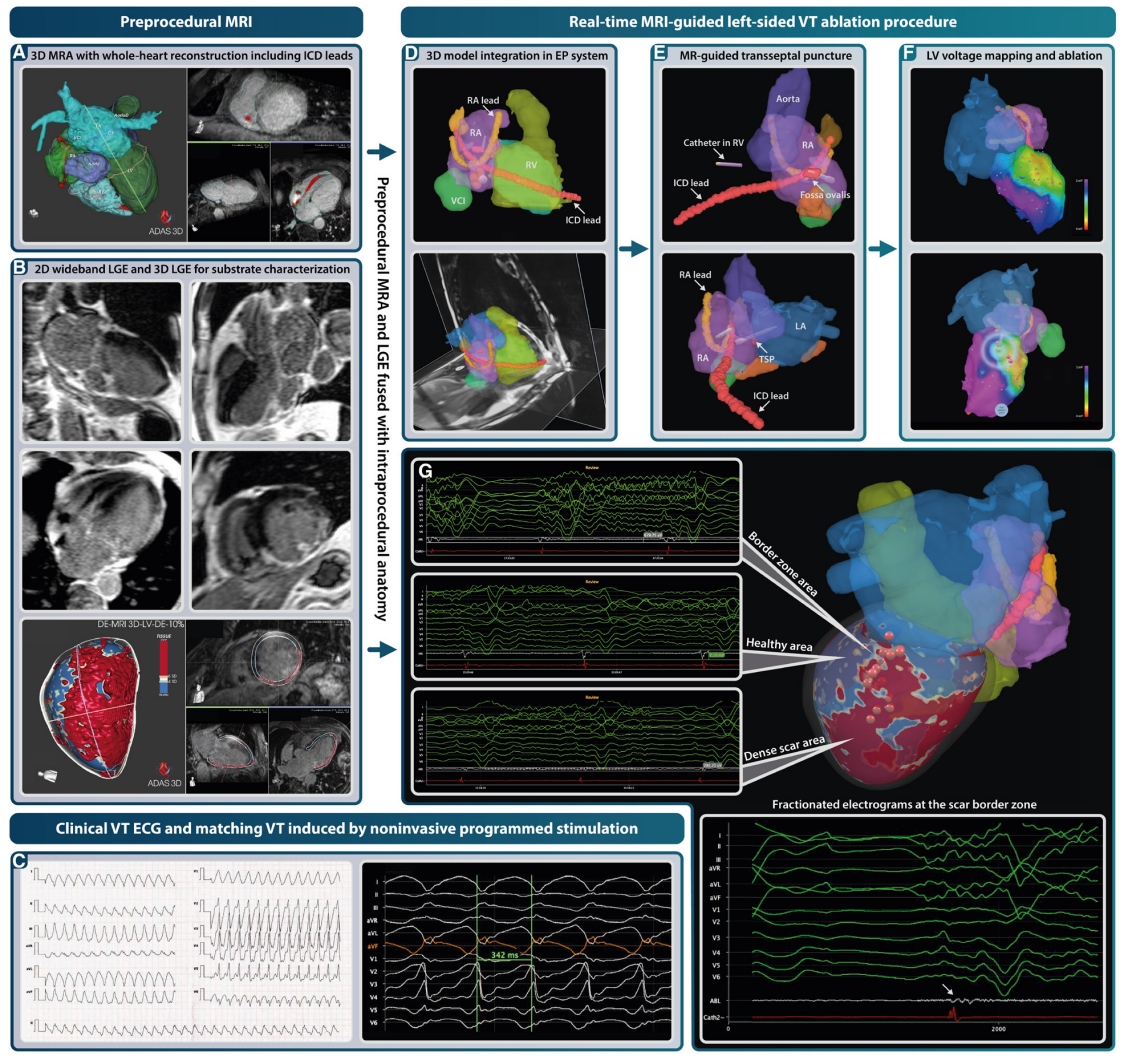
**Overview of the real-time MRI-guided left-sided VT ablation procedure. A**, A diaphragm-navigated 3D contrast-enhanced MR-angiogram with high spatial resolution (in-plane resolution, 1.25×1.25 mm; slice thickness, 2.5 mm; reconstructed to 0.625×0.625×1.25 mm) was segmented into a whole-heart model, with the RA and RV leads delineated to evaluate their course and to plan a septal transseptal trajectory. **B**, Long-axis and short-axis 2D wideband LGE images demonstrating scar in the IPL region. No device-related artifacts on the LGE were visible. A 3D LGE data set of the LV is segmented using ADAS 3D after processing to create a 3D LV scar reconstruction. An anterior artifact (bright signal) from the ICD generator is visible on the 3D LGE images. **C**, Patient’s clinical VT: monomorphic, 173 bpm, extreme axis, right bundle branch block morphology with transition in lead V6. Noninvasive programmed stimulation in the MRI preparation room induced a monomorphic VT consistent with the patient’s clinical arrhythmia (cycle length, 342 ms). **D**, After importing the whole-heart segmentation into the EP navigation and mapping system, the catheters were navigated to the RA and RV using real-time cine overlay. **E**, The transseptal puncture was performed under real-time MR guidance at the predefined fossa ovalis, marked as a red target. Segmentation of the ICD lead facilitated safe catheter navigation. **F**, After reaching the left side of the heart, LV voltage mapping was performed and guided by the 3D LGE LV shell and intracardiac electrograms, VT ablation was undertaken. **G**, Guided by the 3D LGE reconstruction and intracardiac electrical signals, ablation was performed; however, signal processing introduced a time delay between the endocardial electrograms and the surface ECG, and therefore, the RV catheter was used as the reference signal. The border zone LGE region demonstrated late potentials, fractionated electrograms, and a low voltage of 0.68 mV compared with healthy myocardium showing a voltage of 2.19 mV. The dense scar region showed a voltage of 0.28 mV with no capture. 2D indicates 2-dimensional; 3D, 3-dimensional; CMR, cardiac magnetic resonance; DE, delayed enhancement; EP, electrophysiology; ICD, implantable cardioverter-defibrillator; IPL, inferoposterolateral; LGE, late gadolinium enhancement; LV, left ventricle; MRA, magnetic resonance angiogram; RA, right atrium; RV, right ventricle; VCI, vena cava inferior; and VT, ventricular tachycardia.

## PROCEDURAL SETUP

General anesthesia was initiated in the MRI preparation area. Continuous monitoring was maintained using a 12-lead MR-conditional ECG. An investigational MR-conditional external defibrillator was connected throughout the procedure for immediate defibrillation within the scanner if required. Noninvasive programmed stimulation induced a hemodynamically not tolerated monomorphic VT consistent with the clinical arrhythmia (cycle length, 342 ms), which was terminated by antitachycardia pacing (Figure [C]). The ICD was then programmed to MRI mode for the maximum duration of 6 hours.

Arterial pressure monitoring and femoral vascular access were obtained under ultrasound guidance. Two femoral venous sheaths were placed including one for transseptal access. One arterial sheath was placed for potential retrograde left ventricular access. The patient was then transferred to the scanner. The procedure was performed in a 1.5-T scanner adapted for interventional use and connected to a fully integrated MR-conditional electrophysiology platform. A new non-contrast–enhanced 3D MR-angiogram was acquired and segmented to generate an updated whole-heart anatomical model, with the fossa ovalis marked as the transseptal target. Preprocedural 3D magnetic resonance angiography and LGE reconstructions were fused with the intraprocedural model for catheter navigation. Real-time MR-guided catheter visualization with active tracking was used for navigation during transseptal puncture, mapping, and ablation (Figure [D]), primarily relying on the 3D anatomical shell with active catheter tracking. Real-time interactive scanning was used selectively, mainly during initial transseptal orientation and intermittently during the ablation procedure to confirm catheter position and registration of the anatomical model. Patient temperature was continuously monitored throughout the procedure. A low-flow fan was used for patient comfort.

## CATHETER PLACEMENT AND TRANSSEPTAL PUNCTURE

An MR-conditional catheter was positioned in the right ventricle for reference pacing. A large-curve ablation catheter was advanced to the superior vena cava under active MR tracking, guided by segmented anatomy including ICD lead visualization to facilitate septal-side advancement. A long sheath was advanced over the catheter.

At the superior vena cava, the catheter was exchanged for a dilator–needle assembly with active tracking. Controlled pull-back allowed the system to engage the preidentified fossa ovalis, after which transseptal puncture was performed (Figure [E]).

Left atrial access was confirmed by a pressure waveform, and the sheath was advanced into the left atrium. The dilator was removed and the ablation catheter reintroduced.

## MAPPING AND ABLATION

Left ventricular activation and voltage mapping were performed under real-time MR guidance with integration of the 3D LGE model (Figure [F]). The model demonstrated a well-defined inferolateral scar corresponding to low-voltage regions. Late potentials, fractionated electrograms, and low-voltage signals were identified along the lateral scar border (Figure [G]).

Pace mapping showed the best 12-lead match at the basal lateral border, consistent with the presumed exit site. Radiofrequency energy (50 W; ≤60 s) was delivered to sites with abnormal electrograms and the exit region (16 lesions). Loss of local capture confirmed lesion effect, whereas most of the remaining scar was noncapturable, consistent with dense nonconductive tissue.

## TESTING AND FOLLOW-UP

Programmed stimulation induced a nonclinical VT (cycle length, 340 ms) with a more basal exit pattern, prompting delivery of one additional lesion. Subsequent stimulation with up to 3 extra stimuli did not induce sustained VT.

The patient remained in the MRI scanner for approximately 6 hours. The time from scanner entry to first ablation was approximately 4 hours, including imaging with segmentation and alignment, catheter placement, transseptal access, and mapping.

No procedural complications occurred, and the patient remained hemodynamically stable. The ICD was reactivated after the procedure. At 3-month follow-up, no recurrent VT or ICD therapies were documented, and antiarrhythmic therapy was unchanged.

This first-in-human case demonstrates the feasibility of performing left-sided VT ablation entirely under continuous real-time MR guidance. Integration of high-resolution 3D anatomy and LGE enabled accurate catheter tracking, MR-guided transseptal access, direct substrate visualization, and targeted ablation without fluoroscopy.

Early clinical interventional cardiovascular MRI programs focused on typical atrial flutter to establish workflow and safety.^3^ The broader potential of interventional cardiovascular MRI lies in integrating tissue characterization directly into the ablation procedure, an advantage not achievable with conventional electroanatomical mapping. VT ablation, in which substrate definition is central, may represent one of its most impactful applications. In this first-in-human experience, patient selection was guided by VISABL-VT trial inclusion and exclusion criteria, focusing on individuals with postinfarction scar–mediated VT and relatively preserved functional status. Broader adoption in higher-risk populations will require further evaluation of safety measures and procedural strategies.

In this case, LGE accurately delineated the arrhythmogenic substrate, showing close spatial concordance with low voltage, late potentials, and electrogram fractionation. This supported reliable coregistration between MRI-derived tissue information and intracardiac mapping, enabling substrate-guided ablation. Active MR-based tracking provided sufficient precision for lesion delivery and was not degraded by the presence of an ICD or leads, an important consideration.

CMR also offers the potential for intraprocedural visualization of ablation injury, including detection of acute thermal injury and prediction of chronic lesion formation.^5^ Postablation imaging was not performed because the ICD MRI-mode was limited to 6 hours, encompassing both procedural and preparation time, and extension beyond this predefined safety window was not pursued.

Technical challenges remain. Intracardiac electrogram acquisition in the MRI environment is affected by radiofrequency and gradient-induced interference particularly during active scanning, which may reduce signal fidelity during mapping. Surface ECG signals are additionally impacted by magnetohydronamic effects, and signal processing introduced a delay between endocardial and surface recordings; therefore, the RV catheter was used as the timing reference. Current MR-conditional defibrillators allow unsynchronized shocks only, although synchronized capability is anticipated in future systems.

Despite these limitations, this case shows that fully MR-guided workflows can be applied to complex ventricular arrhythmias. Advances in MR-compatible devices, electrogram fidelity, and lesion visualization may further enable precision, radiation-free substrate-directed VT ablation.

## ARTICLE INFORMATION

### Acknowledgments

The authors thank the anesthesiology team for their support during the procedure.

### Disclosures

At the time of the procedure, M.J.W.G. was the principal investigator for the VISABL-VT trial. M.J.B.K. is member of the Imricor Medical Systems advisory board and present principal investigator for the VISABL-VT trial. K.L. is an employee of Imricor Medical Systems, Burnsville, MN. The other authors declared no conflicts of interest. The principal investigator had full access to all the data in the study and takes responsibility for the integrity of the data and the accuracy of the data analysis. The authors confirm that all information and materials presented in the manuscript are original and have not been published or submitted elsewhere.
